# A Genome-Wide Association Study Dissects the Genetic Architecture of the Metaxylem Vessel Number in Maize Brace Roots

**DOI:** 10.3389/fpls.2022.847234

**Published:** 2022-03-10

**Authors:** Meiling Liu, Meng Zhang, Shuai Yu, Xiaoyang Li, Ao Zhang, Zhenhai Cui, Xiaomei Dong, Jinjuan Fan, Lijun Zhang, Cong Li, Yanye Ruan

**Affiliations:** ^1^College of Bioscience and Biotechnology, Shenyang Agricultural University, Shenyang, China; ^2^Key Laboratory of Soybean Molecular Design Breeding, Northeast Institute of Geography and Agroecology, Chinese Academy of Sciences, Changchun, China

**Keywords:** maize (*Zea mays* L.), brace root, metaxylem vessel number, GWAS, candidate gene

## Abstract

Metaxylem vessels in maize brace roots are key tissue, and their number (MVN) affects plant water and inorganic salt transportation and lodging resistance. Dissecting the genetic basis of MVN in maize brace roots can help guide the genetic improvement of maize drought resistance and lodging resistance during late developmental stages. In this study, we used 508 inbred lines with tropical, subtropical, and temperate backgrounds to analyze the genetic architecture of MVN in maize brace roots. The phenotypic variation in MVN in brace roots was evaluated in three environments, which revealed broad natural variation and relative low levels of heritability (*h*^2^ = 0.42). Stiff-stalk lines with a temperate background tended to have higher MVNs than plants in other genetic backgrounds. MVN was significantly positively correlated with plant height, tassel maximum axis length, ear length, and kernel number per row, which indicates that MVN may affect plant morphological development and yield. In addition, MVN was extremely significantly negatively correlated with brace root radius, but significantly positively correlated with brace root angle (BRA), diameter, and number, thus suggesting that the morphological function of some brace root traits may be essentially determined by MVN. Association analysis of MVN in brace roots combined 1,253,814 single nucleotide polymorphisms (SNPs) using FarmCPU revealed a total of nine SNPs significantly associated with MVN at *P* < 7.96 × 10^–7^. Five candidate genes for MVN that may participate in secondary wall formation (*GRMZM2G168365*, *GRMZM2G470499*, and *GRMZM2G028982*) and regulate flowering time (*GRMZM2G381691* and *GRMZM2G449165*). These results provide useful information for understanding the genetic basis of MVN in brace root development. Further functional studies of identified candidate genes should help elucidate the molecular pathways that regulate MVN in maize brace roots.

## Introduction

Water is an important environmental factor with a profound effect on plant growth and development. As a vascular plant species, maize has evolved a xylem vessel system that transports materials such as water and inorganic salts to support plant growth and development. Interestingly, maize brace roots, which are specialized structures, have also developed metaxylem vessels. As an important part of postembryonic roots, brace roots provide water and inorganic substances for maize plants during later stages of growth and development, when seminal roots and primary roots have degraded (Hoppe et al., 1986; Wang and Bai, 2019). In addition, maize brace roots penetrating the soil are inclined to the land, thereby providing the structural support to the plant and strengthening lodging resistance (Hochholdinger et al., 2004; Hochholdinger, 2009). Studies have shown that metaxylem vessels have functions in absorption and support in brace roots (Salvi et al., 2016). The number of metaxylem vessels (MVN) can determine the efficiency of material transport (Liu et al., 2020). In addition, the formation of metaxylem vessels is accompanied by the thickening of secondary cell walls (Turner et al., 2007; Furuta et al., 2014), which are composed of cellulose, hemicellulose and lignin, thus MVN can affect the mechanical strength of brace roots and their structural supporting capacity in maize (Zhao and Dixon, 2011; Oda and Fukuda, 2012; Voxeur et al., 2015; Zhao, 2016). Dissecting the genetic basis of the MVN in brace roots should therefore contribute to the genetic improvement of drought resistance and lodging resistance during late stages of maize development.

The development of brace roots is influenced by multiple factors including heredity and environment. The development of maize brace roots is largely affected by photoperiod (Zhang et al., 2018a), and the same may be true for MVN. The formation of metaxylem vessels is accompanied by two unique biological processes: secondary cell wall deposition and programmed cell death (PCD). The MVN of brace roots might therefore be affected by the activities of enzymes involved in these two biological processes. Many enzymes have been confirmed to participate in metaxylem vessel formation. For example, cellulose synthase (CesA) and phenylalanine ammonia lyase are involved in the biosynthesis of cellulose and lignin (Raes et al., 2003; Taylor et al., 2004), and xylem cysteine proteases are involved in PCD (Avci et al., 2008). Complex regulatory networks including various transcription factors also participate in metaxylem vessels development. In Arabidopsis, NAC transcription factor family members, such as VASCULAR-RELATED NAC-DOMAIN1-7 and NAC SECONDARY WALL THICKENING PROMOTING FACTOR1, act as the master switches of transcription regulatory networks to regulate the expressions of genes involved in secondary cell wall formation and PCD (Zhong et al., 2007; Yamaguchi et al., 2010; Zhou et al., 2014). In addition, some secondary master switches of transcription factors, such as MYB and KNAT7, are regulated by NAC and then control the expressions of downstream functional genes to affect the metaxylem xylem development of roots, stems, and leaves in Arabidopsis (McCarthy et al., 2009; Zhou et al., 2009). Recent studies have shown that maize mutant *necrotic upper tips1* (*nut1*) plants have weak stems and fewer vascular bundles in inflorescence nodes, which results in drought sensitivity and abortion at the flowering stage. Through gene cloning, *NUT1* has been found to encode the NAC transcription factor NAC91, a member of the subfamily of secondary cell wall NAC gene. Mutation of *NAC91* leads to a reduction in xylem vessels and defects in plant water transport (Dong et al., 2020). Despite these findings greatly advanced our understanding of xylem vessel formation, the genetic and molecular mechanisms underlying the regulation of MVN development in maize brace roots are still unclear and need further study.

Genome-wide association study (GWAS) is an effective method to analyze natural variation in quantitative traits on the basis of linkage disequilibrium, thereby providing a theoretical foundation for dissecting the genetic architecture and facilitating the molecular improvement of complex traits in maize (Flint-Garcia et al., 2003; Aranzana et al., 2005; Li et al., 2012). The genetic basis of several maize xylem-related traits has been reported, and some candidate genes have been predicted by the GWAS. For instance, Zhang et al. (2020) used 480 maize inbred lines and 779,855 single nucleotide polymorphisms (SNPs) to conduct a GWAS for vascular bundle number of the third internode of maize stems. They identified 65 unique SNPs, including 5 SNPs identified by multiple methods and distributed on chromosomes 2, 3, 4, 9, and 10. A set of candidate genes associated with vascular bundle number were uncovered to involve signal transduction and stress response (Zhang et al., 2020). GWAS can therefore be used to analyze the genetic basis of MVN in maize brace roots and identify associated candidate genes.

In this study, we used an association panel of 508 maize inbred lines with a wide range of sources and abundant genetic diversity to conduct GWAS of MVN in maize brace roots in three environments. Our aim was to reveal the underlying phenotypic diversity and genetic basis of MVN in maize brace roots. We also analyzed the correlation between MVN and other agronomic traits, including seven morphological traits, seven yield related traits, three maturity traits, and five brace root traits. Using the B73 V2 reference genome, we identified a series of candidate genes associated with brace root MVN. Finally, we analyzed the TPM-based expression levels of these candidate genes in different B73 tissues using information downloaded from the MaizeGDB qTeller database.^1^ The significant SNPs uncovered in this study can be used to dissect the genetic basis of MVN in maize brace roots. In addition, the identified candidate genes can serve as a useful resource for further functional study to improve the transport capacity and lodging resistance of maize during later stages of growth and development.

## Materials and Methods

### Association Mapping Panel

The association panel used for the GWAS contained 508 diverse maize inbred lines. Among them, 60 inbred lines were from the Germplasm Enhancement of Maize in the United States, 223 were from the International Maize and Wheat Improvement Center in Mexico, and 225 were germplasm resources from China. Most of the inbred lines from the International Maize and Wheat Improvement Center were tropical and subtropical, whereas inbred lines from the United States and China were mostly temperate. In a previous study, kinship modeling of the 508 maize inbred lines divided the association panel into four subgroups: 27 stiff stalk (SS), 70 non-stiff stalk (NSS), 196 tropical-subtropical (TST), and 215 admixed (MIXED) lines (Yang et al., 2011). The SS and NSS subgroups encompassed the temperate lines, the TST subgroup comprised tropical and subtropical lines, and the MIXED subgroup contained the remaining non-classified lines. Details on the 508 inbred lines, including population structure, linkage disequilibrium, and genetic diversity have been previously published (Yang et al., 2011; Jiang et al., 2020).

### Field Experiments and Phenotyping

All 508 inbred lines of the association panel were planted in three environments in China: Siping City, Jilin Province in 2017 (JL, 124°35′E, 43°17′N), Shenyang City, Liaoning Province in 2018 (LN, 123°43′E, 41°80′N), and Xinxiang City, Henan Province in 2020 (HN, 113°90′E, 35°30′N). All lines were planted using a randomized complete block design with two replicates. In each plot, each line was planted in a single 2-m long and 0.6-m wide row, with 0.4-m spacing between rows. During the reproductive growth stage, six maize plants of each inbred line were randomly selected. Three brace roots, which were closest to ground level and growing into the soil, were cut with shears. The collected materials were washed with distilled water and placed in a prepared FAA fixative solution for 3–7 days for short-term storage, which softened the roots and made them easily sliceable. Three transverse sections were obtained from each brace root by free-hand sectioning. To clearly visualize metaxylem vessels, the sections were stained with safranin dye, and the MVN of each section was counted under a light microscope (Supplementary Figure 1).

### Statistical Analysis of Phenotypes

A variance analysis and the best unbiased linear predictive value (BLUP) analysis of MVN in maize brace roots were performed under the following linear mixed model in the lme4 package of META-R (Alvarado et al., 2020): *Y*_*ijk*_ = μ + *Rep*_*i*_ + *Gen*_*k*_ + ε_*ijk*_ for individual environment analysis, *Y*_*ijk*_ = μ + *Env*_*i*_ + *Rep*_*j*_(*Env*_*i*_) + *Gen*_*l*_ + *Env*_*i*_×*Gen*_*l*_ + ε_*ijkl*_ for across environments analysis, where *Y*_*ijk*_ is the MVN of interest, μ is the mean effect, *Env*_*i*_ is the effect of the *i*th environment, *Rep_*j*_ (Env_*i*_)* is the effect of the *j*th replicate within the *i*th environment, *Gen*_*l*_ is the effect of the *l*th genotype, *Env*_*i*_ × *Gen*_*l*_ is the environment × genotype interaction, and ε*_*ijk*_* is the error. Broad-sense heritability was, respectively, calculated at individual environment and multiple environments as follows: $h^{2}=\frac{\sigma_{g}^{2}}{\sigma_{g}^{2}+\sigma_{e}^{2}/nreps}$ and $h^{2}=\frac{\sigma_{g}^{2}}{\sigma_{g}^{2}+\sigma_{ge}^{2}/nEnvs+\sigma_{e}^{2}/n\left(Envs\times reps\right)}$, where $\sigma_{g}^{2}$ is the genetic variance, $\sigma_{e}^{2}$ is the residual error, $\sigma_{ge}^{2}$ is the interaction of genotype and environment, and *n* represents the number of environments and replications (Hallauer et al., 2020). For calculating BLUPs and broad-sense heritability, all effects were considered to be random.

### Genome-Wide Association Study

For the GWAS, a genotyping dataset comprising 1,253,814 SNP markers (minor allelic frequency > 5%) obtained from 50K SNP array, 600K SNP array, RNA-Seq, and genotyping-by-sequencing data combined into a whole genetic map was downloaded from www.maizego.org/Resources (Liu et al., 2017). The association analysis for the BLUP value of MVN in three individual environments and across all environments were performed by using the fixed and random model circulating probability unification (FarmCPU) method, which separates a multiple locus linear mixed model into fixed and random effect models to reduce false negatives that can result from a confounding population structure, kinship and SNPs (Cui et al., 2016; Liu et al., 2016). We used a uniform Bonferroni-corrected threshold of α = 1 for the significance cutoff of the linear model as reported in previous studies (Li et al., 2013; Yang et al., 2014; Mao et al., 2015). The suggested *P*-value was thus computed with 1/*n* (*n* = 1,253,814), which resulted in a *P*-value of 7.96 × 10^–7^ for FarmCPU.

The contribution of SNPs to the phenotypic variance was estimated using ANOVA function in the R software package. After adjustment for population structure effects, the *R*^2^ of each significant SNP was calculated using two linear models: *Y* = *X*_*i*_α_*i*_ + *P*β + ε, which was used to estimate the total variance of all significant SNPs, and *Y* = *X*_α_ + *P*β + ε, which was used to estimate the variance of individual significant SNPs. In these equations, *Y* and *X* represent phenotype and SNP genotype vectors, respectively, *P* is the matrix of the three subgroups (NSS, SS, TST), and α, β, and ε are SNP, subgroup and random effects, respectively.

### Annotation of Candidate Genes

The most significant SNP was chosen to represent the locus in the same LD block (*r*^2^ < 0.2). The physical locations of SNPs were determined in reference to the B73 RefGen_v2 genome. Genes within a 50-kb range upstream and downstream of the significantly associated SNP locus were searched (Cui et al., 2020; Jiang et al., 2020), and functionally annotated using information on homologous *Arabidopsis thaliana* and rice genes. Genes with homologs related to photoperiod, secondary cell wall synthesis and PCD were designated as candidate genes for MVN.

### Heat Map of Candidate Genes

The expression levels of candidate genes from different maize tissues of B73 were downloaded from the MaizeGDB qTeller database (see text footnote 1). The values used for heat map construction were calculated as log_10_ (n + 1), where *n* is the TPM value.

## Results

### Metaxylem Vessels Diversity and Heritability

According to our analysis of phenotypic variation, the MVN of brace roots in the association panel exhibited extensive phenotypic variation that followed a normal distribution (Supplementary Figure 2). The broad-sense heritability (*h*^2^) of MVN ranged from 0.43 in the across environments analysis to 0.74 in the JL environment (Table 1). Variance analysis of MVN indicated that the effect of genotypic variance was highly significant (*P* < 0.01) in single environments and across all environments, while the genotype × environment (G × E) interaction was highly significant (*P* < 0.01) for across environments but environment variance was not significant.

**TABLE 1:** Variance composition and broad-sense heritability of metaxylem vessel number (MVN) in maize brace roots in the association panel with three individual environments and across all environments.

Env^a^	Means ± SD	Range^b^	Variance component^c,d^			*h* ^2e^
			Genotype (G)	Environment (E)	G × E	
JL	40.17 ± 5.59	26.93–58.71	43.48**	−	−	0.74
LN	36.01 ± 6.94	27.30–50.31	25.98**	−	−	0.61
HN	37.12 ± 3.50	33.09–48.03	15.76**	−	−	0.49
All	37.55 ± 5.15	32.85–45.52	10.82**	0.74	24.56**	0.43

*^a^JL Jilin Province, LN Liaoning Province, HN Henan Province, All across three environments.*

*^b^The range of BLUP value of metaxylem vessel number in three individual environments and across all environments.*

*^c^G and E indicated genotype and environment, respectively, and G × E indicated interaction of G and E.*

^d^** Significant at P ≤ 0.01.

*^e^Broad-sense heritability.*

**FIGURE 1:**
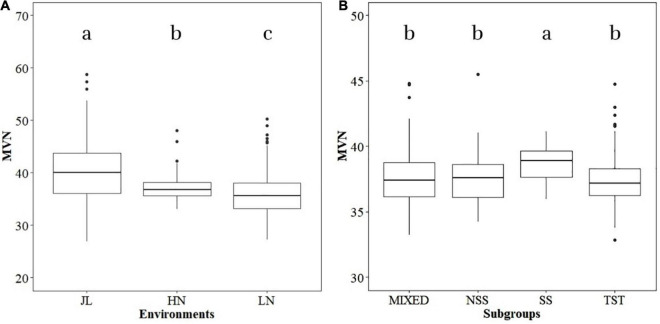
Boxplot of metaxylem vessel number (MVN) in maize brace roots distribution in different environments and subgroups. Analysis of variance (ANOVA) was applied to examine the difference of phenotypes among different environments and subgroups. Different letters indicate statistically significant differences at *P* ≤ 0.05. **(A)** Different environments, **(B)** different subgroups.

The phenotypic variance of MVN in different environments (JL, LN, and HN) was analyzed independently. Significant differences were detected in MVN among the three environments (Figure 1A). The median of MVN was 39.99 in JL and significantly higher than that in HN (36.81) and LN (35.62), and the variation range of the MVN in JL was also more extensive than LN and HN. The phenotypic variation of MVN was also compared among different subgroups to investigate the effect of population structure (Figure 1B). The median MVN in the SS subgroup was significantly higher than that in other subgroups. In addition, the range of phenotypic variation of MVN was smaller in the SS subgroup than in other subgroups. In summary, MVN exhibited broad variation according to genetic backgrounds and environments.

### Correlations of Metaxylem Vessels With Other Plant Developmental Processes

**FIGURE 2:**
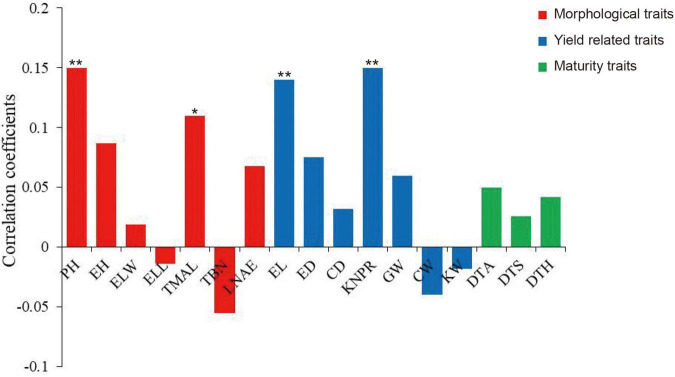
Correlation coefficients of metaxylem vessel number (MVN) in maize brace roots with 17 other agronomic traits based on BLUP values. *, significant at *P* ≤ 0.05; **, significant at *P* ≤ 0.01. PH, plant height; EH, ear height; ELW, ear leaf width; ELL, ear leaf length; TMAL, tassel maximum axis length; TBN, tassel branch number; LNAE, leaf number above ear; EL, ear length; ED, ear diameter; CD, cob diameter; KNPR, kernel number per row; CGW, cob grain weight; CW, cob weight; KW, kernel width; DTA, days to anthesis; DTS, days to silking; DTH, days to heading.

Because the MVN of maize brace roots can affect plant growth and development by influencing the transport of water and inorganic substances, we investigated the correlations between MVN and other agronomic traits. Pearson’s correlation coefficients were calculated after comparison of MVN with 17 agronomic traits previously measured in the same association panel, including seven morphological traits: plant height (PH), ear height (EH), ear leaf width (ELW), ear leaf length (ELL), tassel maximum axis length (TMAL), tassel branch number (TBN), and leaf number above ear (LNAE), seven yield traits: ear length (EL), ear diameter (ED), cob diameter (CD), kernel number per row (KNPR), cob grain weight (CGW), cob weight (CW), kernel width (KW), and three maturity traits: days to anthesis (DTA), days to silking (DTS), and days to heading (DTH) (Yang et al., 2014). MVN was extremely significantly positively correlated with PH, EL, and KNPR, and significantly positively correlated with TMAL, thus indicating that MVN can affect plant morphology and yield (Figure 2).

**FIGURE 3:**
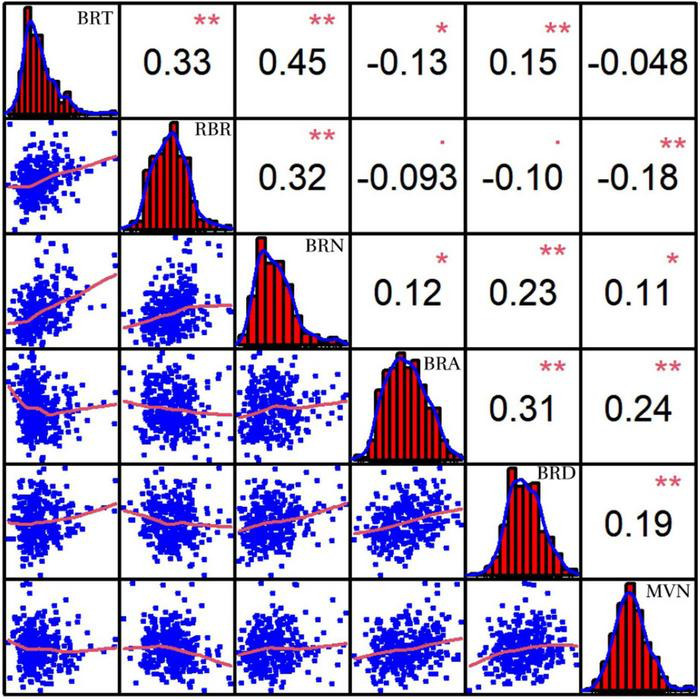
Correlation coefficients of metaxylem vessel number (MVN) in maize brace roots with five other brace root traits based on BLUP values. BRT, brace root tier number; RBR, brace root radius; BRN, brace root number; BRA, brace root angle; BRD, brace root diameter, *, significant at *P* ≤ 0.05; **, significant at *P* ≤ 0.01.

In addition, MVN can also affect plant lodging resistance by influencing the mechanical strength of brace roots. Pearson’s correlation coefficients were also calculated after comparison of MVN with five brace root morphological traits: brace root tier number (BRT), brace root radius (RBR), brace root number (BRN), brace root angle (BRA), and brace root diameter (BRD) (Unpublished results), which can affect plant yield and lodging resistance (Reneau et al., 2020). MVN was extremely significantly negatively correlated with RBR. MVN was extremely significantly positively correlated with BRA and BRD, and significantly positively correlated with BRN (Figure 3).

### Genome-Wide Association Study

**FIGURE 4:**
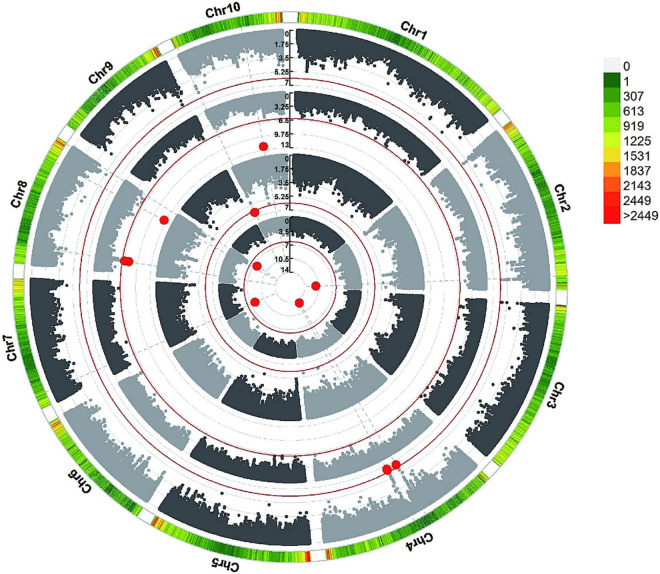
GWAS-derived Manhattan plots showing significant SNPs associated with metaxylem vessel number (MVN) in maize brace roots using FarmCPU. Each dot represents a SNP. The red solid line represents the Bonferroni-corrected significance threshold of 7.96 × 10^–7^. From the inside to the outside: LN, JL, HN, and across all environments.

To reduce the impact of environmental and repetitive variation, phenotypic BLUP values across all three environments and in three individual environments (JL, LN, and HN) were used for the association study. The GWAS was performed using the FarmCPU method with a threshold of *P* < 7.96 × 10^–7^. We detected four independent significant SNPs above this threshold on chromosomes 2, 4, 7, and 8 in LN, which accounted for 4.88–6.72% of phenotypic variation and 30.00% of total phenotypic variation (Table 2, Figure 4, and Supplementary Figure 3). Three independent significant SNPs were identified on chromosomes 8 and 10 in HN, they explained 1.23–5.93% of phenotypic variation and 11.76% of total phenotype variation (Table 2 and Figure 4). Only one independent significant SNP accounting for 6.26% of phenotype variation was identified on chromosome 10 in JL. Two independent significant SNPs above this threshold on chromosome 4 were detected in across three environments and accounted for 5.80 and 11.92% of phenotype variation, respectively, and 13.99% of total phenotype variation (Table 2 and Figure 4). An identical SNP, chr4s-96312065, was detected in both the LN environment and across three environments analysis. This SNP was located in the third exon of *GRMZM2G168365*, which encoded sugars will eventually be exported transporter 15a (SWEET15a) protein (Supplementary Table 2 and Figure 4).

**TABLE 2:** The independent SNP and chromosomal positions significantly associated with metaxylem vessel number (MVN) in maize brace roots identified by GWAS using the FarmCPU method.

Environment	SNP	Chr	Position (bp)	Allele^a^	*P*-value	*R* ^2b^
LN	chr2.S_218589725	2	218,589,725	G/A	6.61E-12	5.29
	chr4.S_96312065	4	96,312,065	C/T	8.56E-14	6.72
	Chr7.S_18230705	7	18,230,705	C/T	7.56E-09	4.88
	Chr8.S_174297913	8	174,297,913	G/A	1.43E-07	5.73
Total^c^						30.00
HN	chr8.S_25018483	8	25,018,483	G/C	2.21E-07	1.23
	chr8.S_145474885	8	145,474,885	T/C	2.64E-07	5.14
	chr10.S_94253633	10	94,253,633	C/T	6.53E-07	5.93
Total^c^						11.76
JL	chr10.S_2987778	10	2,987,778	C/T	4.77E-07	6.26
Total^c^						6.26
Across	chr4.S_96312065	4	96,312,065	C/T	3.82E-07	5.80
	chr4.S_114281801	4	114,281,801	G/A	2.89E-07	11.92
Total^c^						13.99

*^a^Major/minor allele; underlined bases indicate favorable alleles.*

*^b^Percentage of phenotypic variation explained by the additive effect of the single significant SNP.*

*^c^Total percentage of phenotypic variation explained by all significant SNPs.*

**FIGURE 5:**
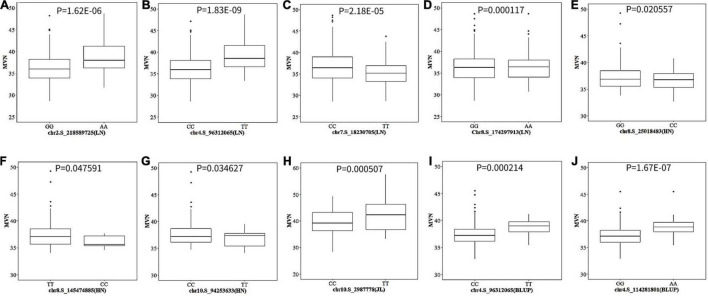
The boxplot of phenotypic difference between the major alleles and minor alleles for significant SNPs associated with metaxylem vessel number (MVN) in maize brace roots. The *P*-values (Student’s *t*-test) of the allelic effects of MVN are exhibited above each small plot. **(A–J)** Ten significant SNPs associated with MVN in maize brace roots with three individual environments and across all environments.

The allele effects of these 10 significant SNPs were also evaluated (Figure 5). The phenotypic distribution differences for MVN between the major alleles and minor alleles were extremely significant for all 10 significant SNPs. The allele effects of chr4.S_96312065 (LN) on chromosome 4 was the most significant for MVN phenotypic variation with *P*-value of 1.83E-09, which was consistent with FarmCPU results (Figure 5).

### Genetic Overlap of Metaxylem Vessels With Aerial Root Number and Flowering Time

**FIGURE 6:**
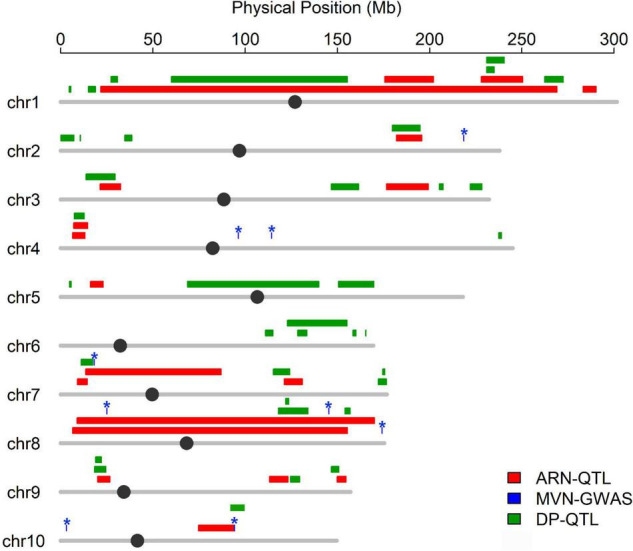
Co-localization between the significant SNPs associated with metaxylem vessel number in brace roots and QTL for aerial nodal root number and day to pollen. Colored lines represent QTL region for different traits, The asterisk represents the significant SNPs associated with metaxylem vessel number.

Flowering time plays an important role in shaping the nodal root number via indirect selection during maize domestication (Zhang et al., 2018a), which inspired us to further explore whether MVN had overlap with nodal root number and flowering time. The positional information on QTLs for aerial nodal root number (ARN) and day to pollen (DP) were downloaded, and DP was used for evaluating flowering time (Zhang et al., 2018a). Three significant SNPs associated with MVN had overlap with QTLs for ARN, and one significant SNP associated with MVN had overlap with QTL for DP (Figure 6). There had no overlap between these three traits, but significant SNPs Chr7.S_18230705 on chromosomes 7, which had overlap with ARN, was close to the QTL of DP, and chr10.S_94253633 on chromosomes 10, which had overlap with DP, was close to the QTL of ARN in physical distance (Figure 6), therefore the same candidate genes were expected to be found for these three traits.

### The Annotation and Expression Pattern of Candidate Genes

**FIGURE 7:**
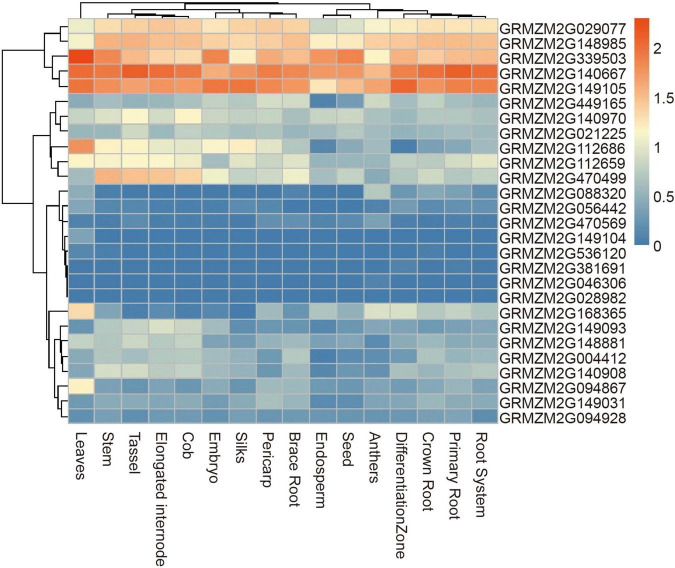
Heat map of expression patterns of candidate genes identified by GWAS in different tissues. The values used in the figure were the log10 (n + 1) transformed of normalized TPM valve. Columns and rows were ordered according to similarity (hierarchical cluster analysis at the top and left). The red and blue represented higher and lower expression level in different tissues, respectively.

A total of 28 genes were found within a 50-kb range upstream and downstream of the significant SNPs. Of these genes, 23 have been functionally annotated (Supplementary Table 2). According to the functional annotation, the 23 genes were mainly divided into four functional types: metabolic, material transport, transcription regulation, and cellulose biosynthesis. To further determine whether these genes had function in plants, their expression patterns were analyzed using published RNA-Seq datasets from 16 different tissues, including brace roots (Figure 7). 19 genes were expressed in most tissues with no tissue-specificity, possibly because vessels were present in most tissues. *GRMZM2G029077*, *GRMZM2G148985*, *GRMZM2G339503*, *GRMZM2G140667*, and *GRMZM2G149105* had higher expression tendencies relative to other genes.

The candidate genes of four significant SNPs that had overlap with QTLs for ARN and DP were firstly analyzed. Nine genes were found, and only one gene, *GRMZM2G381691*, was reported to control the nodal root number and flowering time, so it was presumed to be candidate genes for MVN in brace roots (Table 3). Additionally, according to the functional annotation, *GRMZM2G449165* were presumed to be candidate genes for MVN in brace roots and might be involved in regulating flowering time, and *GRMZM2G168365*, *GRMZM2G470499*, *GRMZM2G028982* were presumed to be candidate genes for MVN in brace roots, and might be involved in secondary cell wall formation, which was necessary processes for the formation of metaxylem vessels.

**TABLE 3:** The candidate genes of four significant SNP that had overlap with QTLs for aerial nodal root number or day to pollen.

SNP	Chr	Position	Candidate gene	Anotation	QTLs for ARN^a^	QTLs for DP^b^	Selection^c^
Chr7.S_18230705	7	18,230,705	GRMZM2G094928	VMA10	Y	N	U
			GRMZM2G094867	LEA			U
chr8.S_25018483	8	25,018,483	GRMZM2G046306	GDSL	Y	U	U
			GRMZM2G112686	GDSL			U
			GRMZM2G112659	VIT	Y	U	U
chr8.S_145474885	8	145,474,885	GRMZM2G028982	OFP34			U
			GRMZM2G029077	AP4B			U
			GRMZM2G339503	Tom7			U
chr10.S_94253633	10	94,253,633	GRMZM2G381691	CCT1	N	Y	Y

*^a^ARN aerial nodal root number, Y represented the SNP overlapped with QTL for ARN; N represented the SNP was close to QTL for ARN; U represented no overlap between SNP and QTL for ARN.*

*^b^DP day to pollen, Y represented the SNP overlapped with QTL for DP, N represented the SNP was close to QTL for DP, U represented no overlap between SNP and QTL for DP.*

*^c^Y represented that the candidate gene was selected by flowering time. U signified that the candidate gene was not selected by flowering time.*

## Discussion

The reported brace root-related traits in maize, such as BRN, radius, and tier number all show extensive phenotypic variation, the heritability are medium to high levels (Zhang et al., 2018b). In the present study, brace-root MVN also exhibited a wide range of phenotypic variations and followed a normal distribution. According to our genetic analysis, the heritability of MVN was 0.43 with a relatively low level, indicating that it was controlled by multiple small-effect genes. The genotype effects for MVN were significant, thus indicating that MVN was mainly influenced by genetic factors and suitable for further GWAS analysis. A phenotypic variation analysis uncovered significant differences in MVN among different environments, which suggested that different environments could enhance or diminish the effects of MVN variance. Variance analysis of MVN indicated that the effect of G × E interaction on the MVN was highly significant, which was consistent with the observed phenotypic variation in different environments. Different lines can therefore be selected for specific environments to improve MVN in maize breeding programs, and eight identified environmental specific significant SNPs may play crucial roles in MVN development.

Maize was originated and domesticated in tropical regions and was then grown and improved in subtropical and temperate regions. The morphological structure of maize is therefore strongly controlled by the population structure (Camus-Kulandaivelu et al., 2006). To identify the effect of population structure on the MVN of brace roots, we compared MVN phenotypic variation between different maize subgroups (Figure 1). The brace roots of temperate SS maize lines tend to have more metaxylem vessels. Previous studies had found that the root architecture with more brace roots and fewer crown roots are favored in temperate maize, which efficiently improve root-lodging resistance and the ability to uptake water and nitrogen (Zhang et al., 2018a). The transport tissue of brace roots is metaxylem vessels, Thus, more MVN might also be selected in temperate maize. Furthermore, the development of maize brace roots is largely affected by photoperiod, and the selection for flowering time has indirectly reshaped the root system (Zhang et al., 2018a). Among identified nine significant SNPs, one significant SNP chr10.S_94253633 had overlap with QTL of DP, and was close to the QTL of ARN in physical distance, and one significant SNP Chr7.S_18230705 was close to the QTL of DP, which had overlap with QTL of ARN. Therefore, flowering time might also have an indirectly role for shaping MVN in brace root during maize domestication.

The development of brace roots is affected by photoperiod. *GRMZM2G381691* was presumed to be candidate genes for MVN in brace roots, which was reported to control the nodal root number and flowering time (Zhang et al., 2018a). *GRMZM2G381691* encoded ZmCCT1 which were identified to be associated with flowering time by QTL mapping and association mapping and led to early flowering (Hung et al., 2012; Yang et al., 2013). In addition, the crown root number was more significantly accumulated in the *ZmCCT1* transgenic plants than the non-transgenic plants (Zhang et al., 2018a). Therefore, *GRMZM2G381691* may affect MVN indirectly by regulating flowering time during maize domestication. Moreover, the candidate gene *GRMZM2G449165* related to MVN, detected in LN in our study, encodes VASCULAR PLANT ONE-ZINC FINGER 5 (VOZ5), which was highly homologous with VOZ1 in Arabidopsis. AtVOZ proteins mediate the first step of the phyB signal transduction pathway to regulate flowering, *voz1voz2* mutant suppressed the expression of *FT*, and repressed the early flowering phenotype of the *phyB* mutant (Yasui et al., 2012). *GRMZM2G449165* may thus regulate MVN in maize brace roots by regulating flowering time.

Furthermore, metaxylem vessel formation mainly involves some biological processes including cell division, cell specialization, secondary wall thickening and PCD (Milioni et al., 2001). In this study, functional annotation revealed that three candidate genes, which were involved in secondary wall formation, could regulate metaxylem vessel formation. The deposition of secondary cell walls, in which cellulose, hemicellulose and lignin are deposited in annular, spiral, reticulate or pitted patterns to provide mechanical support for the plant, is an essential process in metaxylem vessel formation. Many genes involved in these processes, including cellulose synthase- and lignin monomer synthesis-related genes, have been identified in Arabidopsis. A series of *CesA* mutants is found to have irregular xylem, which cause a collapse of mature xylem cells in the inflorescence stems of Arabidopsis (Turner and Somerville, 1997; Taylor et al., 2003). The candidate gene *GRMZM2G470499*, related to MVN in the LN environment, encodes CesA14, which directly determines cellulose synthesis (Taylor et al., 1999, 2007; Paredez et al., 2006). *GRMZM2G028982* may therefore affect MVN in brace roots by participating in secondary cell wall formation. The candidate gene *GRMZM2G168365*, which was identified in the LN environment and across environments analysis, encodes sugars will eventually be exported transporter15a (SWEET15a) protein. SWEET proteins participate in the transport of hexose and sucrose in plants (Chardon et al., 2013; Guo et al., 2013; Yuan and Wang, 2013), and are involved in many processes such as plant reproductive development (Guan et al., 2008), leaf senescence (Seo et al., 2011), and abiotic stress response (Chen et al., 2015; Eom et al., 2015) in Arabidopsis. Subsequently, transcriptome analysis revealed that the expressions of *SWEET16b* and *SWEET1a* are higher in the Saccharum leaf source-sink transition zone, where cell activities are mainly focused on tetrapyrrole, lignin and secondary cell wall biosynthesis, thus suggesting that SWEET16b and SWEET1a may play a role in secondary cell wall biosynthesis (Hu, 2017). *GRMZM2G168365* may therefore influence MVN in maize brace roots by affecting secondary cell wall formation.

A transcriptional regulatory pathway in which NAC transcription factors act as the main regulatory switch to control other downstream transcription factors or functional proteins to regulate secondary cell wall formation and PCD has also been identified in Arabidopsis (Zhong et al., 2007; Zhou et al., 2014). The candidate gene *GRMZM2G028982* related to MVN, detected in HN, encodes OVATE-transcription factor 34 (OFP34). Interfascicular fiber wall thickness is increased in the basal internodes of the *ofp4* mutant and *ofp1ofp4* double mutant in Arabidopsis, similar to *knat7* mutants (Zhong et al., 2008; Li et al., 2011). KNAT7 is one of the direct targets of SND1 (belong to NAC family) and MYB46, which thereby regulate secondary wall biosynthesis (Ko et al., 2009). Subsequent studies have shown that KNAT7 can interact with OFP1 and OFP4 to enhance the transcriptional inhibitory activity of KNAT7, which helps regulate secondary cell wall formation in Arabidopsis (Li et al., 2011). Consequently, *GRMZM2G028982* may regulate MVN in brace roots by affecting secondary cell wall formation.

## Conclusion

In this study, we have revealed the genetic architecture of natural variation in MVN in maize brace roots by the GWAS approach. MVN showed a relatively low heritability and showed broad variation in the association panel used in this investigation. According to the GWAS, several genetic loci with small effects regulate the natural variation in MVN in maize brace roots, and flowering time might have an indirectly role for shaping MVN in brace roots during maize domestication. Five candidate genes were detected that possibly impact MVN by affecting flowering time and secondary wall formation. These candidate genes are a valuable resource for further studies to dissect the molecular network regulating MVN in maize brace roots. In addition, the significantly associated SNPs found in this study should be helpful in facilitating marker assisted selection of appropriate brace-root MVNs in maize breeding programs.

## Data Availability Statement

The original contributions presented in the study are included in the article/Supplementary Material, further inquiries can be directed to the corresponding author/s.

## Author Contributions

CL and YR initiated and designed the overall study. XL and MZ performed and coordinated the field experiments and phenotypic data collection. ML and MZ carried out the data analysis. ML interpreted the results and wrote the manuscript. All authors contributed to manuscript editing.

## Conflict of Interest

The authors declare that the research was conducted in the absence of any commercial or financial relationships that could be construed as a potential conflict of interest.

## Publisher’s Note

All claims expressed in this article are solely those of the authors and do not necessarily represent those of their affiliated organizations, or those of the publisher, the editors and the reviewers. Any product that may be evaluated in this article, or claim that may be made by its manufacturer, is not guaranteed or endorsed by the publisher.

## Footnotes

1
https://qteller.maizegdb.org/

## Supplementary Material

Supplementary Figure 1 The phenotypic images of MVN in HN environment.

Supplementary Figure 2 Frequency distributions of metaxylem vessel number (MVN) of maize brace roots in association panel. The red line represented the frequency distribution curve of MVN, whereas the blue line represented the standard normal distribution curve. **(A)** LN environment; **(B)** JL environment; **(C)** HN environment; **(D)** across all environments.

Supplementary Figure 3 GWAS-derived QQ plots using FarmCPU. **(A)** LN environment; **(B)** JL environment; **(C)** HN environment; **(D)** across all environments.

## Abbreviations

MVN: metaxylem vessel number
PCD: programmed cell death
CesA: cellulose synthase
GWAS: genome-wide association study
PH: plant height
EH: ear height
ELW: ear leaf width
ELL: ear leaf length
TMAL: tassel maximum axis length
TBN: tassel branch number
LNAE: leaf number above ear
EL: ear length
ED: ear diameter
CD: cob diameter
KNPR: kernel number per row
CGW: cob grain weight
CW: cob weight
KW: kernel width
DTA: days to anthesis
DTS: days to silking
DTH: days to heading
BRT: brace root tier number
RBR: brace root radius
BRN: brace root number
BRA: brace root angle
BRD: brace root diameter
ARN: aerial nodal root number
DP: day to pollen
JL: Jilin
LN: Liaoning
HN: Henan.

## References

[B1] Alvarado G. Rodríguez F. M. Pacheco A. Burgueño J. Crossa J. Vargas M. (2020). META-R: a software to analyze data from multi-environment plant breeding trials. *Crop J.* 5 745–756. 10.1016/j.cj.2020.03.010

[B2] Aranzana M. J. Kim S. Zhao K. Bakker E. Horton M. Jakob K. (2005). Genome-wide association mapping in Arabidopsis identifies previously known flowering time and pathogen resistance genes. *PLoS Genet.* 1:e60. 10.1371/journal.pgen.0010060 16292355 PMC1283159

[B3] Avci U. Petzold H. E. Ismail I. O. Beers E. P. Haigler C. H. (2008). Cysteine proteases XCP1 and XCP2 aid micro-autolysis within the intact central vacuole during xylogenesis in Arabidopsis roots. *Plant J.* 56 303–315. 10.1111/j.1365-313X.2008.03592.x 18573193

[B4] Camus-Kulandaivelu L. Veyrieras J. B. Madur D. Combes V. Fourmann M. Barraud S. (2006). Maize adaptation to temperate climate: relationship between population structure and polymorphism in the Dwarf 8 gene. *Genetics* 172 2449–2463. 10.1534/genetics.105.048603 16415370 PMC1456379

[B5] Chardon F. Bedu M. Calenge F. Klemens P. A. W. Spinner L. Clement G. (2013). Leaf fructose content is controlled by the vacuolar transporter SWEET17 in Arabidopsis. *Curr. Biol.* 23 697–702. 10.1016/j.cub.2013.03.021 23583552

[B6] Chen H. Y. Huh J. H. Yu Y. C. Ho L. H. Chen L. Q. Tholl D. (2015). The Arabidopsis vacuolar sugar transporter SWEET 2 limits carbon sequestration from roots and restricts Pythium infection. *Plant J.* 83 1046–1058. 10.1111/tpj.12948 26234706

[B7] Cui Z. H. Dong H. X. Zhang A. Ruan Y. Y. Zhang Z. W. (2020). Denser markers and advanced statistical method identified more genetic loci associated with husk traits in maize. *Sci. Rep.* 10:8165. 10.1038/s41598-020-65164-0 32424146 PMC7235265

[B8] Cui Z. H. Luo J. H. Qi C. Y. Ruan Y. Y. Li J. Zhang A. (2016). Genome-wide association study (GWAS) reveals the genetic architecture of four husk traits in maize. *BMC Genom.* 17:946. 10.1186/s12864-016-3229-6 27871222 PMC5117540

[B9] Dong Z. B. Xu Z. N. Xu L. Galli M. Chuck G. (2020). Necrotic upper tips1 mimics heat and drought stress and encodes a protoxylem-specific transcription factor in maize. *Proc. Natl. Acad. Sci.* 117 20908–20919. 10.1073/pnas.2005014117 32778598 PMC7456077

[B10] Eom J. S. Chen L. Q. Sosso D. Julius B. T. Lin I. W. Qu X. Q. (2015). SWEETs, transporters for intracellular and intercellular sugar translocation. *Curr. Opin. Plant Biol.* 25 53–62. 10.1016/j.pbi.2015.04.005 25988582

[B11] Flint-Garcia S. A. Thornsberry J. M. Buckler E. S. (2003). Structure of linkage disequilibrium in plants. *Ann. Rev. Plant Biol.* 54 357–374. 10.1146/annurev.arplant.54.031902.134907 14502995

[B12] Furuta K. M. Hellmann E. Helariutta Y. (2014). Molecular control of cell specification and cell differentiation during procambial development. *Ann. Rev Plant Biol.* 65 607–638. 10.1146/annurev-arplant-050213-040306 24579995

[B13] Guan Y. F. Huang X. Y. Zhu J. Gao J. F. Zhang H. X. Yang Z. N. (2008). Ruptuerd Pollen Grain11, a member of the MtN3/saliva gene family, is crucial for exine pattern formation and cell integrity of microspore in Arabidopsis. *Plant Physiol.* 147 852–863. 10.1104/pp.108.118026 18434608 PMC2409014

[B14] Guo W. J. Nagy R. Chen H. Y. Pfrunder S. Yu Y. C. Santelia D. (2013). SWEET17, a facilitative transporter, mediates fructose transport across the tonoplast of Arabidopsis roots and leaves. *Plant Physiol.* 164 777–789. 10.1104/pp.113.232751 24381066 PMC3912105

[B15] Hallauer A. R. Carena M. J. Miranda-Filho J. B. (2020). *Quantitative Genetics in Maize Breeding.* New York, NY: Springer.

[B16] Hochholdinger F. (2009). “The Maize Root System: Morphology, Anatomy, and Genetics,” in *Handbook of Maize: Its Biology*, eds Bennetzen J. L. Hake S. C. (New York, NY: Springer), 145–160. 10.1007/978-0-387-79418-1_8

[B17] Hochholdinger F. Park W. J. Sauer M. Woll K. (2004). From weeds to crops: genetic analysis of root development in cereals. *Trends Plant Sci.* 9 42–48. 10.1016/j.tplants.2003.11.003 14729218

[B18] Hoppe D. C. McCully M. E. Wenzel C. L. (1986). The nodal roots of Zea: their development in relation to structural features of the stem. *Can. J. Bot.* 64 2524–2537. 10.1139/b86-335

[B19] Hu W. C. (2017). *The Evolution and Function of SWEET Genes in Saccharum (in Chinese).* China: Fujian agriculture and Forestry University.

[B20] Hung H. Y. Shannon L. M. Tian F. Bradbury P. J. Chen C. Flint-Garcia S. A. (2012). ZmCCT and the genetic basis of day-length adaptation underlying the postdomestication spread of maize. *Proc. Natl. Acad. Sci.* 109 1913–1921. 10.1073/pnas.1203189109 22711828 PMC3396540

[B21] Jiang S. Q. Zhang H. B. Ni P. Z. Yu S. Dong H. X. Zhang A. (2020). Genome-Wide Association Study Dissects the Genetic Architecture of Maize Husk Tightness. *Front. Plant Sci.* 11:861. 10.3389/fpls.2020.00861 32695127 PMC7338587

[B22] Ko J. H. Kim W. C. Han K. H. (2009). Ectopic expression of MYB46 identifies transcriptional regulatory genes involved in secondary wall biosynthesis in Arabidopsis. *Plant J.* 60 649–665. 10.1111/j.1365-313X.2009.03989.x 19674407

[B23] Li E. Y. Wang S. C. Liu Y. Y. Chen J. G. Douglas C. J. (2011). OVATE FAMILY PROTEIN4 (OFP4) interaction with KNAT7 regulates secondary cell wall formation in Arabidopsis thaliana. *Plant J. Cell Mol. Biol.* 67 328–341. 10.1111/j.1365-313X.2011.04595.x 21457372

[B24] Li H. Peng Z. Y. Yang X. H. Wang W. D. Fu J. J. Wang J. H. (2013). Genome-wide association study dissects the genetic architecture of oil biosynthesis in maize kernels. *Nat Gen.* 45 43–50. 10.1038/ng.2484 23242369

[B25] Li Q. Yang X. Xu S. Cai Y. Zhang D. Han Y. (2012). Genome-wide association studies identified three independent polymorphisms associated with α-tocopherol content in maize kernels. *PLoS One* 7:e36807. 10.1371/journal.pone.0036807 22615816 PMC3352922

[B26] Liu H. J. Xin L. Niu L. (2017). Distant eQTLs and Non-coding Sequences Play Critical Roles in Regulating Gene Expression and Quantitative Trait Variation in Maize. *Mol. Plant* 10 414–426. 10.1016/j.molp.2016.06.016 27381443

[B27] Liu X. L. Huang M. Fan B. Buckler E. S. Zhang Z. W. (2016). Iterative Usage of Fixed and Random Effect Models for Powerful and Efficient Genome-Wide Association Studies. *PloS Gen.* 12:e1005767. 10.1371/journal.pgen.1005767 26828793 PMC4734661

[B28] Liu Z. Hartmann A. Hajirezaei M. R. Carpentier S. Wirén N. (2020). Seminal and nodal roots of barley differ in anatomy, proteome and nitrate uptake capacity. *Plant Cell Physiol*. 7 1297–1308. 10.1093/pcp/pcaa059 32379871

[B29] Mao H. D. Wang H. W. Liu S. X. Li Z. G. Yang X. H. Yan J. B. (2015). A transposable element in a NAC gene is associated with drought tolerance in maize seedlings. *Nat. Commun.* 6:8326. 10.1038/ncomms9326 26387805 PMC4595727

[B30] McCarthy R. L. Zhong R. Ye Z. H. (2009). MYB83 is a direct target of SND1 and acts redundantly with MYB46 in the regulation of secondary cell wall biosynthesis in Arabidopsis. *Plant Cell Physiol.* 50 1950–1964. 10.1093/pcp/pcp139 19808805

[B31] Milioni D. Sado P. E. Stacey N. J. Domingo C. Roberts K. McCann M. C. (2001). Differential expression of cell-wall-related genes during the formation of tracheary elements in the Zinnia mesophyll cell system. *Plant Mol. Biol.* 47 221–238. 10.1023/A:1010647902487 11554474

[B32] Oda Y. Fukuda H. (2012). Secondary cell wall patterning during xylem differentiation. *Curr. Opin. Plant Biol.* 15 38–44. 10.1016/j.pbi.2011.10.005 22078063

[B33] Paredez A. R. Somerville C. R. Ehrhardt D. W. (2006). Visualization of Cellulose Synthase Demonstrates Functional Association with Microtubules. *Science* 312 1491–1495. 10.1126/science.1126551 16627697

[B34] Raes J. Rohde A. Christensen J. H. Boerjan P. W. (2003). Genome-wide characterization of the lignification toolbox in Arabidopsis. *Plant Physiol.* 133 1051–1071. 10.1104/pp.103.026484 14612585 PMC523881

[B35] Reneau J. W. Khangura R. S. Stager A. Erndwein L. Weldekidan T. Cook D. D. (2020). Maize brace roots provide stalk anchorage. *Plant Direct.* 4:e00284. 10.1002/pld3.284 33204937 PMC7649601

[B36] Salvi S. Giuliani S. Ricciolini C. Carraro N. Maccaferri M. Presterl T. (2016). Two major quantitative trait loci controlling the number of seminal roots in maize co-map with the root developmental genes rtcs and rum1. *J. Exp. Bot.* 67 1149–1159. 10.1093/jxb/erw011 26880748 PMC4753855

[B37] Seo P. J. Park J. M. Kang S. K. Kim S. G. Park C. M. (2011). An Arabidopsis senescence-associated SAG29 regulates cell viability under high salinity. *Planta* 233 189–200. 10.1007/s00425-010-1293-8 20963606

[B38] Taylor N. G. Gardiner J. C. Whiteman R. Turner S. R. (2004). Cellulose synthesis in the Arabidopsis secondary cell wall. *Cellulose* 11 329–338. 10.1023/B:CELL.0000046405.11326.a8

[B39] Taylor N. G. Gardiner J. C. Whiteman R. Turner S. R. (2007). Cellulose Synthesis in the Arabidopsis Secondary Cell Wall. *Cellulose* 11 329–338. 10.1007/978-1-4020-5380-1_4

[B40] Taylor N. G. Howells R. M. Huttly A. K. Vickers K. Turner S. R. (2003). Interactions among three distinct cesa proteins essential for cellulose synthesis. *Proc. Natl. Acad. Sci.* 100 1450–1455. 10.1073/pnas.0337628100 12538856 PMC298793

[B41] Taylor N. G. Scheible W. R. Cutler S. Somerville C. R. Turner S. R. (1999). The irregular xylem3 Locus of Arabidopsis Encodes a Cellulose Synthase Required for Secondary Cell Wall Synthesis. *Plant Cell* 11 769–779. 10.1105/tpc.11.5.769 10330464 PMC144224

[B42] Turner S. Gallois P. Brown D. (2007). Tracheary element differentiation. *Ann. Rev. Plant Biol.* 58 407–433. 10.1146/annurev.arplant.57.032905.105236 17472568

[B43] Turner S. R. Somerville C. R. (1997). Collapsed xylem phenotype of Arabidopsis identifies mutants deficient in cellulose deposition in the secondary cell wall. *Plant Cell* 9 689–701. 10.2307/3870425 9165747 PMC156949

[B44] Voxeur A. Wang Y. Sibout R. (2015). Lignification: different mechanisms for a versatile polymer. *Curr. Opin. Plant Biol.* 23 83–90. 10.1016/j.pbi.2014.11.006 25449731

[B45] Wang C. Bai Y. (2019). Maize aerial roots fix atmospheric N2 by interacting with nitrogen fixing bacteria (in Chinese). *Sci. Sin. Vitae.* 49 89–90.

[B46] Yamaguchi M. Kubo M. Fukuda H. Demura T. (2010). Vascular-related NAC-domain7 is involved in the differentiation of all types of xylem vessels in Arabidopsis roots and shoots. *Plant J.* 55 652–664. 10.1111/j.1365-313X.2008.03533.x 18445131

[B47] Yang N. Lu Y. L. Yang X. H. Huang J. Zhou Y. Ali F. (2014). Genome Wide Association Studies Using a New Nonparametric Model Reveal the Genetic Architecture of 17 Agronomic Traits in an Enlarged Maize Association Panel. *PLoS Gen.* 10:e1004573. 10.1371/journal.pgen.1004573 25211220 PMC4161304

[B48] Yang Q. Li Z. Li W. Q. Ku L. X. Wang C. Ye J. R. (2013). CACTA-like transposable element in ZmCCT attenuated photoperiod sensitivity and accelerated the postdomestication spread of maize. *Proc. Natl. Acad. Sci. U.S.A.* 110 16969–16974. 10.1073/pnas.1310949110 24089449 PMC3801022

[B49] Yang X. H. Gao S. B. Xu S. T. Zhang Z. X. Prasanna B. M. Li L. (2011). Characterization of a global germplasm collection and its potential utilization for analysis of complex quantitative traits in maize. *Mol. Breed.* 28 511–526. 10.1007/s11032-010-9500-7

[B50] Yasui Y. Mukougawa K. Uemoto M. Yokofuji A. Suzuri R. Nishitani A. (2012). The phytochrome-interacting vascular plant one-zinc finger1 and VOZ2 redundantly regulate flowering in Arabidopsis. *Plant Cell* 24 3248–3263. 10.2307/41692798 22904146 PMC3462629

[B51] Yuan M. Wang S. P. (2013). Rice MtN3 / Saliva / SWEET family genes and their homologs in cellular organisms. *Mol. Plant* 6 665–674. 10.1093/mp/sst035 23430047

[B52] Zhang Y. Wang J. L. Du J. J. Zhao Y. X. Lu X. J. Wen W. L. (2020). Dissecting the phenotypic components and genetic architecture of maize stem vascular bundles using high-throughput phenotypic analysis. *Plant Biotechnol. J.* 19 35–50. 10.1111/pbi.13437 32569428 PMC7769239

[B53] Zhang Z. H. Zhang X. Lin Z. L. Wang J. Xu M. L. Lai J. S. (2018a). The genetic architecture of nodal root number in maize. *Plant J.* 93 1032–1044. 10.1111/tpj.13828 29364547

[B54] Zhang A. Cui Z. H. Li C. Luo J. H. Guan Y. X. Liu L. L. (2018b). Identification of maize brace-root quantitative trait loci in a recombinant inbred line population. *Euphytica* 214:168. 10.1007/s10681-018-2203-6

[B55] Zhao Q. (2016). Lignification: flexibility, biosynthesis and regulation. *Trends Plant Sci.* 21 713–721. 10.1016/j.tplants.2016.04.006 27131502

[B56] Zhao Q. Dixon R. A. (2011). Transcriptional networks for lignin biosynthesis: more complex than we thought. *Trends Plant Sci.* 16 227–233. 10.1016/j.tplants.2010.12.005 21227733

[B57] Zhong R. Lee C. Zhou J. L. Mccarthy R. L. Ye Z. H. (2008). A battery of transcription factors involved in the regulation of secondary cell wall biosynthesis in Arabidopsis. *Plant Cell* 20 2763–2782. 10.1105/tpc.108.061325 18952777 PMC2590737

[B58] Zhong R. Richardson E. A. Ye Z. H. (2007). The MYB46 transcription factor is a direct target of SND1 and regulates secondary wall biosynthesis in Arabidopsis. *Plant Cell* 19 2776–2792. 10.1105/tpc.107.053678 17890373 PMC2048704

[B59] Zhou J. Lee C. Zhong R. Ye Z. H. (2009). MYB58 and MYB63 are transcriptional activators of the lignin biosynthetic pathway during secondary cell wall formation in Arabidopsis. *Plant Cell* 21 248–266. 10.1105/tpc.108.063321 19122102 PMC2648072

[B60] Zhou J. Zhong R. Ye Z. H. (2014). Arabidopsis NAC domain proteins, VND1 toVND5, are transcriptional regulators of secondary wall biosynthesis in vessels. *PLoS One* 9:e105726. 10.1371/journal.pone.0105726 25148240 PMC4141820

