# Supplementary figures and images for: A Genome-Wide Association Study Dissects the Genetic Architecture of the Metaxylem Vessel Number in Maize Brace Roots
Source: Front Plant Sci. 2022 Mar 10;13:847234. doi: 10.3389/fpls.2022.847234 (PMC8961028; doi:10.3389/fpls.2022.847234)

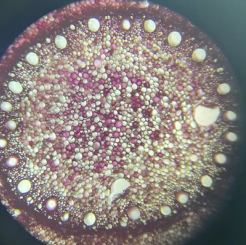

MVN=23 (BY843)

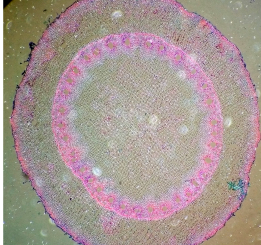

MVN=34 (CIMBL125)

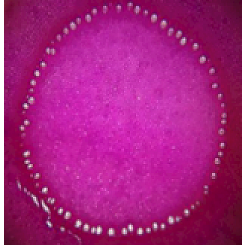

MVN=65 (JH59)

Supplement: Supplementary Figure 1 — The phenotypic images of MVN in HN environment. [file Image_1.pdf]

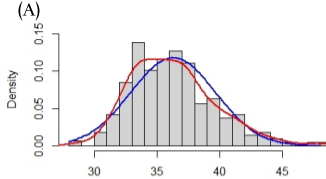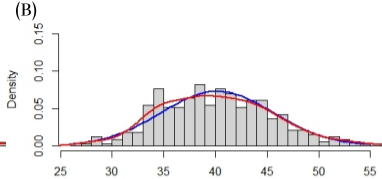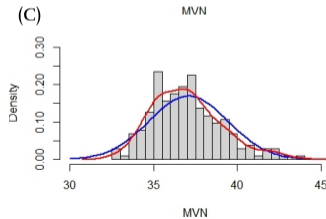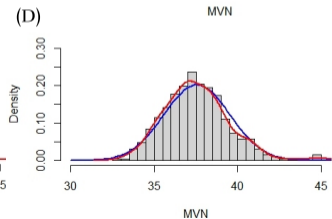

Supplement: Supplementary Figure 2 — Frequency distributions of metaxylem vessel number (MVN) of maize brace roots in association panel. The red line represented the frequency distribution curve of MVN, whereas the blue line represented the standard normal distribution curve. (A) LN environment; (B) JL environment; (C) HN environment; (D) across all environments. [file Image_2.pdf]

(A)

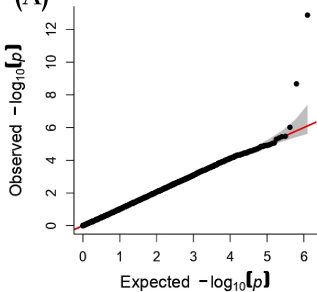

(B)

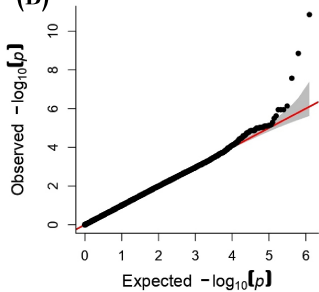

(C)

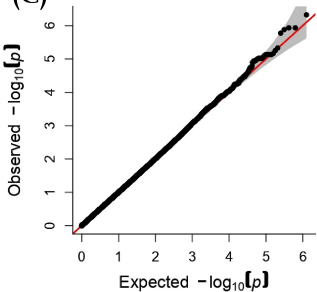

(D)

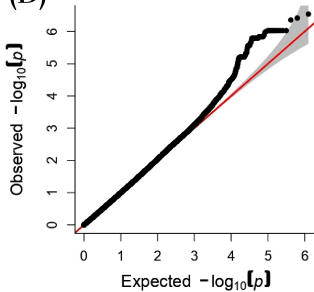

Supplement: Supplementary Figure 3 — GWAS-derived QQ plots using FarmCPU. (A) LN environment; (B) JL environment; (C) HN environment; (D) across all environments. [file Image_3.pdf]
